# Primary cilia: The central role in the electromagnetic field induced bone healing

**DOI:** 10.3389/fphar.2022.1062119

**Published:** 2022-11-29

**Authors:** Yangmengfan Chen, Chao Lu, Xifu Shang, Kerong Wu, Kun Chen

**Affiliations:** ^1^ Department of Orthopedics, West China Hospital, West China School of Medicine, Sichuan University, Chengdu, China; ^2^ Department of Hand and Foot Surgery, The Affiliated Hospital of Qingdao University, Qingdao, China; ^3^ Department of Orthopedics, The First Affiliated Hospital of USTC, Division of Life Sciences and Medicine, University of Science and Technology of China, Hefei, China

**Keywords:** electromagnetic fields, primary cilia, osteogenesis, signaling pathway, bone

## Abstract

Primary cilia have emerged as the cellular “antenna” that can receive and transduce extracellular chemical/physical signals, thus playing an important role in regulating cellular activities. Although the electromagnetic field (EMF) is an effective treatment for bone fractures since 1978, however, the detailed mechanisms leading to such positive effects are still unclear. Primary cilia may play a central role in receiving EMF signals, translating physical signals into biochemical information, and initiating various signalingsignaling pathways to transduce signals into the nucleus. In this review, we elucidated the process of bone healing, the structure, and function of primary cilia, as well as the application and mechanism of EMF in treating fracture healing. To comprehensively understand the process of bone healing, we used bioinformatics to analyze the molecular change and associated the results with other studies. Moreover, this review summarizedsummarized some limitations in EMFs-related research and provides an outlook for ongoing studies. In conclusion, this review illustrated the primary cilia and related molecular mechanisms in the EMF-induced bone healing process, and it may shed light on future research.

## 1 Introduction

The skeletal system is the framework that not only provides the architectural stability of the connective tissues but also protects the organs. Therefore, bone fractures caused by injury, osteoporosis, cancer, or other systemic diseases are one of the most serious diseases (De Mattos et al., 2012). Considering there are 7.9 million fractures in the United States annually, with the increase in disabilities and the prolonged recovery time, bone fractures exacerbate heavy socioeconomic and healthcare burdens (Victoria et al., 2009). Although lots of research and great progress have been made in this field, 5%–10% of fractures worldwide still developed into delayed-union or nonunion (Claes et al., 2012). Thus, further research for improving the clinical outcomes is urgently necessary.

For thoroughly exploring the treatment of fracture healing, the physiological and pathological activities of bone should be clarified first. In general, the skeletal system is a dynamic and highly coordinated system, which involves the balance of both osteoblastic bone formation and osteoclastic bone resorption (Kaku and Komatsu, 2017). After the fracture, the healing process follows a regular timeline: 1) Intramembranous ossification: within the first 5 days, mesenchymal stem cells (MSCs) and osteoprogenitors migrate to the fracture site, then they proliferate and differentiate at the subperiosteal callus region. 2) Endochondral ossification: during days 5–9, transforming growth factor-β (TGF-β), osteocalcin (OCN), and various cytokines are released to promote chondrocytes proliferation. 3) During days 9–14, the chondrocytes become mature, the proliferation decreases, and even undergo apoptosis gradually. But the osteogenesis sustains in this process, the soft callus is mineralized with matrix deposition, the woven bone is generated, the vascular invasion is initiated gradually. 4) Between days 14–21, the new bone is regenerated actively. 5) After day 21, cellular proliferation ceases, the bone remodeling process initiates (Daish et al., 2018).

**FIGURE 1:**
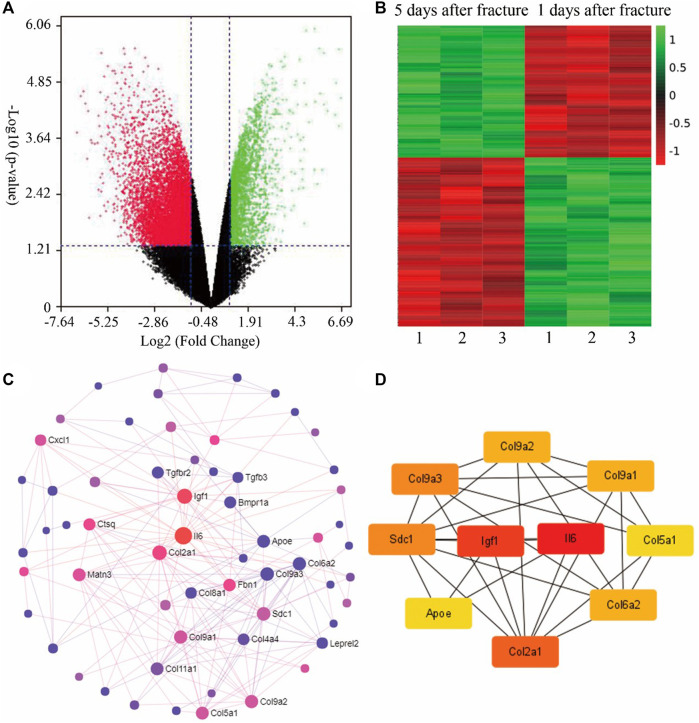
Bioinformatics analysis of fracture calluses at 1-day and 5-day post fracture of young mice (GSE17825). **(A)** The volcano plot for the gene expression in both groups. **(B)** According to the *p*-values, the heat map includes the expression of the top 250 differentially expressed genes of both groups. **(C)** The protein-protein interaction (PPI) of the top 100 genes generated by String and Network Analyst. **(D)** The top 10 hub genes analyzed by Cytoscape, *i.e.*, Igf1, Il6, Col9a2, Col9a3, Sdc1, Apoe, Col2a1, Col6a2, and Col5a1. Col9a1 (figures were plotted by https://www.bioinformatics.com.cn (last accessed on 30 July 2022), an online platform for data analysis and visualization).

To observe the change in gene expression during fracture healing, gene expression datasets (GSE17825) of fracture was analyzed from the Gene Expression Omnibus (GEO) database (Khan et al., 2008). Gene expression analysis was performed on the groups of 1 day and 5 days post fracture of young mice. The volcano plot indicated that there is a total of 13,661 genes with significant difference between 1 and 5 days post fracture (Figure 1A). The expression of top 250 genes is shown in the heat map, which confirmed that gene expression is clustered by timepoint, and the differences can be observed (Figure 1B). To discern the relationship between the top 250 genes, the STRING database was used to analyse the protein-protein interactions (PPI) (Figure 1C). Then the top 10 hub genes were determined by the Cytoscape software (Figure 1D), which demonstrated that the processes of inflammation, extracellular matrix, and cartilage development are involved.

However, during the healing process, there are various biochemical and biophysical stimuli, such as fluid shear, microgravity, as well as the electromagnetic field, which can affect cellular activities and the clinical outcomes. Therefore, it is promising and worthwhile to explore, optimize, and modulate these physiochemical signals to positively promote bone fractures. To reach this goal, several prerequisites should be fulfilled by applying physiochemical signals to affect cellular activities: 1) there should exist organelles which can perceive these extracellular signals, 2) the specific translator that can translate these signals into the biochemical messages, 3) an effective signalingsignaling pathway which can transmit this information to the nucleus (Temiyasathit and Jacobs, 2010). Therefore, we focused on the cellular “antenna”—primary cilium. In this review, we intend to delineate the key role of and the molecular mechanism of the primary cilium in the process of bone healing.

## 2 The structure and function of the primary cilium

The primary cilium is a microtubule-based non-motile organelle that protrudes from the membrane in nearly all eukaryotic cells. Compared with motile cilium, primary cilium lacks the two central microtubules (9 + 0 structure) and other axonemal components, such as radial spokes and dynein arms (Schwartz et al., 1997). Primary cilium can detect, transmit, and interpret physiochemical signals from the local environment into cells to regulate proliferation, migration, and differentiation (Prevo et al., 2017). This function owe to the ciliary membrane that contains lots of receptors that are associated with various signalingsignaling pathways (Seeger-Nukpezah and Golemis, 2012).

Primary cilium cannot synthesize proteins for themselves, so the existence of a transport system between primary cilia and the cell body is necessary for delivering necessary proteins for genesis and maintenance of the cilium. In 1993, Rosenbaum *et al.* verified this transport system by finding the bidirectional movement of particles in the space between the membrane of flagellar and the doublet microtubules (Kozminski et al., 1993). This orderly movement is the intraflagellar transport (IFT) system, which is a complex and highly regulated cellular activity initiated immediately after the early stage of ciliogenesis and operated by various IFT proteins and motors (Taschner et al., 2012). The IFT proteins are required for the structure and function of the primary cilium. IFT proteins can be classified into two types, and functions as retrograde or anterograde transporting proteins between cell body and primary cilium (Table 1). Nevertheless, the components of the IFT-B complex, both IFT25 and IFT27, can also deliver the proteins of hedgehog (Hh) signaling through the retrograde IFT (Keady et al., 2012; Eguether et al., 2014). IFT 140 was highly related to the osteogenic ability given that the ageing and ovariectomy (OVX) induced osteoporosis models expressed significantly lower IFT 140 (Zhang C. Y. et al., 2019). Further study showed that the targeted deletion of IFT 80 (Yuan et al., 2016) or IFT 88 (Tummala et al., 2010) resulted in impaired osteogenic ability. Recently, primary cilium was proven to be an important organelle to promote bone healing by reacting and transducing extracellular physiochemical signals, such as electromagnetic fields (EMFs).

**TABLE 1:** The classification and function of the intraflagellar transport (IFT) system.

Transporting direction	IFT subtypes	Components of IFT proteins	Motor protein
retrograde transport: from the cilium to the cell body	IFT-A	IFT 43, IFT 121, IFT 122, IFT 139, IFT 140, and IFT 144	dynein-2
anterograde transport: from the cell body to the cilium	IFT-B Core subcomplex	IFT 22, IFT 25, IFT 27, IFT 46, IFT 52, IFT 56, IFT 70, IFT 74, IFT 81, and IFT 88/Polaris	kinesin-2
anterograde transport: from the cell body to the cilium	IFT-B Peripheral subcomplex	IFT 20, IFT 38, IFT 54, IFT 57, IFT 80, and IFT 172	kinesin-2

## 3 The development of electromagnetic fields in bone healing

In 1978, Bassett *et al.* successfully applied pulsed electromagnetic fields (PEMFs) in treating bone fractures (Bassett et al., 1978). After that, the United States Food and Drug Administration (FDA) approved PEMF as a kind of safe and effective physical treatment for osteoporosis, delayed union, and nonunion fractures. Nowadays, it has been widely recognized that PEMFs can bring tremendous clinical benefits because of its noninvasive property, safety, low-cost, home implementation, and none-adverse effects (Yuan et al., 2018).

To better understand the potential mechanism, it is necessary to elucidate the character of EMFs. EMF exists everywhere in our daily life and can be categorized into different types depending on the origin (from permanent magnets/electric currents), production (natural/man-made), status (static/dynamic), and ionization ability (ionizing/non-ionizing radiation). In the field of regenerative medicine, EMFs with specific frequency receive substantial attention. Based on the frequency, EMFs can be classified into: 1) extremely low-frequency EMFs (ELF-EMFs, < 300 Hz), 2) intermediate frequency EMFs (300 Hz–10 MHz), and 3) radiofrequency EMFs (10 MHz–300 GHz) (Wang and Zhang, 2017). Interestingly, electric fields (EFs) and magnetic fields (MFs) are different because Efs exert electronic forces on charged molecules, the cell membrane, and the cellular organelles directly (Ross et al., 2015). Thus, Efs can mediate the cell migration by directly regulating the dynamics of microfilament and microtubules (Finkelstein et al., 2004). MFs can penetrate through the cell, influent the biochemical reaction, and regulate protein synthesis (Funk et al., 2009). Therefore, MFs may play a more important role in stimulating bone cells that were surrounded by tissues and bone. From numerous types of EMFs, the ELF-PEMFs combine various advantages, which can generate a series of magnetic pulses, and each magnetic pulses can also generate an electrical signal in turn (Goodman and Shirley-Henderson, 1990). Most importantly, ELF-EMFs were proven to be the non-ionizing, low-energy, and significant influences on the cellular and subcellular activities (Bassett, 1993).

The generation of EMFs whichs in accord with Faraday’s Lawwhichch declared that EFs or MFs are generated when electrons move at a constant velocity, while EMFs are generated when electrons move at an accelerated velocity, and the change of EFs or MFs can generate each other mutually (Ross et al., 2015). Therefore, it is theoretically feasible to regulate the endogenous EMFs at the wound site with the exogenous EMFs device (Ross et al., 2015). Yasuda *et al.* reported that physiological EMFs exist in the human body because the piezoelectric phenomenon (The classic, 1977). The further study verified that the physiological frequency of EMFs during the movement of the musculoskeletal system ranges from 5 to 30 Hz (Antonsson and Mann, 1985). Under pathological conditions, Becker et al. (1962) reported that during the healing process, the differences in electric potentials around the wound would gradually decline to zero. This phenomenon could be explained by the endogenous EFs formation between the lower electric potential in the wounded site, and the higher electric potential in the healthy tissue; thus, the transepithelial potential difference (TEP) is generated in the local wound (Song et al., 2002). Therefore, EMFs provide lots of benefits for wound healing and are a potential treatment for fracture healing.

## 4 The function of primary cilia in transducing electromagnetic field signals to promote bone healing

Primary cilia of osteoblast cells were first discovered by Tonna and Lampen, (1972). After that, the fundamental function of primary cilia in bone metabolism attracted extensive attention. Osteocytes, a subset of osteoblasts located in the “lacunae”, play an important role in transferring the extracellular mechanical signals into the bone to regulate the balance of osteogenesis and osteoclastogenesis. Primary cilium of osteocyte works as: 1) most osteocytes stay in G0/G1 stage, so they exhibit matured primary cilia (Kaku and Komatsu, 2017); 2) The primary cilium of osteocyte touches the lacunar tightly because the length of primary cilia (2–9 μm) is much longer than the lacunae space (0.1–3 μm) (Jacobs et al., 2010); 3) After receiving extracellular signals by primary cilia, osteocytes can communicate with surrounding cells through the gap junction (Jacobs et al., 2010). Therefore the length of the primary cilium is most important for its function for two reasons: 1) if the length of primary cilia is too short to touch the wall of the “lacunae”, the osteocytes cannot perceive mechanical forces, and 2) longer primary cilia have a larger surface area, more receptors, ion channels, thus are more sensitive to extracellular signals (Yuan and Yang, 2016).

Primary cilia are indispensable in transducing extracellular signals. For example, under the treatment of fluid flow, osteocyte cell lines MLO-Y4 showed excellent osteogenic ability (Malone et al., 2007). However, after damaging primary cilia by using the chloral hydrate or IFT88 siRNA, the expression of OPG/RANKL decreased (Malone et al., 2007), and the osteogenesis was impaired (Tummala et al., 2010). The polycystin-1 (PC1), a component of primary cilium that plays a key role in mechanotransduction, PC1 knockdown reduced the expression of both the early and late osteogenic genes, such as runt-related transcription factor 2 (Runx2), OCN, and osterix (OSX) (Wang et al., 2014). Thus, the methods to increase the length of primary cilia is an effective treatment to promote osteogenesis, such as suppressing of adenylate cyclase III by lithium, inhibiting the anterograde direction IFT *via* deletion of Dynlt1 (Palmer et al., 2011), and regulating the actin cytoskeleton by using actin polymerization inhibitor cytochalasin D (Shi et al., 2017), as well as EMFs treatment.

**FIGURE 2:**
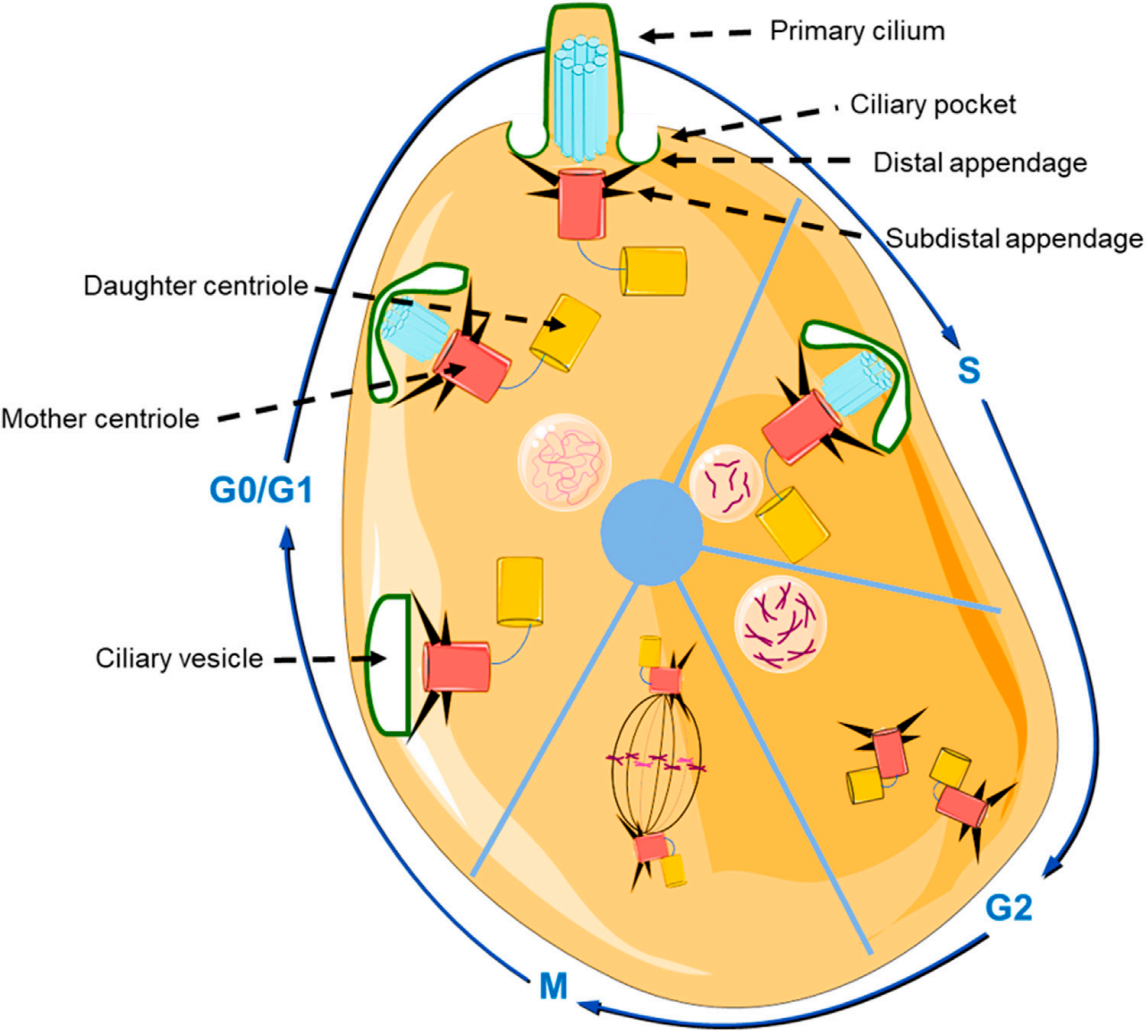
Schematic representation of the structure of the primary cilium, and the relationship between cell cycle and primary ciliogenesis. In the beginning of G0/G1 phase, the centrosome moves beneath the cell membrane and subsequently initiates ciliogenesis and gradually forms the primary cilium. In S phase and G2 phase, primary cilium is degraded, and the centrioles are duplicated and prepare to transform into the mitotic spindle pole in the M phase (The graphic components of the figure were provided by smart.servier.com).

To shed light on primary cilia related osteogenesis, it is necessary to mention the interesting relationship between ciliogenesis and the cell cycle (Figure 2). Primary cilia can be formed *via* either extracellular way or intracellular way (Ghossoub et al., 2011). Firstly, in the extracellular pathway, the centrioles will move beneath the cell membrane, and transform to the basal body (BB). Then, the BB will bind to the cell membrane, after that the axonemal microtubules are formed. In contrast, during the intracellular pathways, the BB will dock to the Golgi derived ciliary vesicles (CV) firstly, then the axoneme elongates, and the CV fused with the plasm membrane (Huber et al., 1993).

The BB plays a central role during the genesis of primary cilia. The BB is a cylindrical organelle that consists of nine symmetrically triplet microtubules and membranes, and the formation of BB is regulated by the cell cycle (Figure 2). During the G0/G1 phase, the centrosome moves beneath the cell membrane, the mother centriole that was used for axoneme nucleation transforms into the BB and initiates ciliogenesis (Kobayashi and Dynlacht, 2011). Then, primary cilia start to be degraded in the G2 phase and S phase. This phenomenon occurs because the centrioles are duplicating and preparing to form the mitotic spindle pole in the M phase (Ford et al., 2018). In addition, there is a ciliary transition zone (TZ) that exists in the space between the BB and the primary cilium. The special structure of TZ consists of Y-links, transition fibers, and a ciliary necklace, that not only functions as a physical boundary that can separate primary cilia from the cytoplasm (Hildebrandt et al., 2011), but also exhibits an indispensable role in controlling the movement of proteins by only selecting proteins of proper size (∼10 nm) (Kee et al., 2012). Those proteins larger than 10 nm accumulate at the ciliary pocket (CiPo) instead of entering into cilia (Deane et al., 2001). Therefore, it is plausible that CiPo involves in transducing or amplifying of extracellular signals toward primary cilia (Benmerah, 2013).

Moreover, the cell cycle can also be regulated by the length of primary cilia. It was reported that an IFT88 knockdown promoted hASCs proliferation (Bodle et al., 2013). The loss of Nde1, which is a centrosome protein, leaded to the longer primary cilia, and retarded cell cycle re-entry (Kim and Tsiokas, 2011). In addition, the phosphorylation of a dynein subunit Tctex-1 resulted in disassembly of the primary cilia and maintenance in S phase (Li et al., 2011).

Due to the relationship between the cell cycle and primary cilia, the serum starvation is used to promote the assembly of primary cilia by arresting cells in the G0 phase. In contrast, serum stimulation is used to disassemble primary cilia by promoting cell cycle re-entry. It was reported the influence of serum stimulation on the disassembly of primary cilia takes effect immediately after 1–2 and 18–24 h of treatment (Pugacheva et al., 2007). Moreover, PEMF (15 Hz, 0.5 mT) can also regulate the cell cycle to inhibit apoptosis of osteocyte-like cells in the presence of primary cilia in an intensity-dependent manner (Wang et al., 2019). Altogether, considering that ciliogenesis initiates at the interphase, the cell proliferation and differentiation/ciliogenesis should be mutually exclusive. Therefore, the presence of primary cilia could be a maker to distinguish the cell cycle and differentiation.

## 5 The mechanism between ELF-PEMFs, primary cilia, and bone fracture

Recently, the potential mechanism of ELF-PEMFs induced bone healing has received more attention. It was reported that ELF-PEMFs (26 Hz) could regulate the level of various proteins in hMSCs, such as Akt, Erk 1/2, Hsp27, and p53, in a frequency and time-dependent exposure manner (Poh et al., 2018). Therefore, it is plausible that ELF-PEMFs can enhance osteogenesis by modulating specific signaling pathways, such as TGF-β/BMP signaling, Hedgehog (Hh) signaling, mitogen-activated protein kinase (MAPK) signaling, platelet-derived growth factor (PDGF) signaling, Wnt signaling, and calcium ion channel. In this review, the major purpose is to clarify the interaction between primary cilia, ELF-PEMFs, and TGF-β/BMP signaling.

### 5.1 TGF-β and BMP signaling pathways

The TGF-β superfamily contains TGF-βs, bone morphogenic proteins (BMPs), activins (ACTs), and inhibins (INHs), from which both TGF-βs and BMPs are most outstanding in regulating the bone healing. TGF-β is a potent chemotactic stimulator that plays numerous key functions in the development and maintenance of bone system, such as cell migration, proliferation, osteogenic differentiation, and matrix mineralizationmineralization (Tsiridis et al., 2007). Typically, TGF-βs are classified into TGF-β1, TGF-β2, and TGF-β3. They share highly homologous amino acid sequences with each other (Yue and Mulder, 2001). Without the stimulation, TGF-β connects with the matrix to maintain an inactive status, under the stimulation of some bioactive molecules, such as matrix metalloproteinases (MMPs), BMPs, or reactive oxygen species (ROS), the inactive TGF-β will be released from the matrix, and transform to an active TGF-β to play various biological functions (Horiguchi et al., 2012).

The TGF-β receptor (TGF-β RI/ALK5 and TGF-β RII) is located on the surface of the ciliary membrane, which is a heterotetrameric receptor of serine/threonine kinases (Clement et al., 2013). TGF-β RII, in the activated state after binding with ligands, will phosphorylate TGF-β RI. In canonical signaling, TGF-β RI will then phosphorylate Smad2/3. The p-Smad2/3 will bind to Smad4 to enter into the cell nucleus to regulate cellular activities. Besides, TGF-βs also involve in many non-canonical signaling pathways, such as NF-κB signaling (Luo, 2017) and ERK/MAPK signaling (Clement et al., 2013), at the ciliary base. In 2013, Clement et al. (2013) proved that both TGF-β RI and TGF-β RII, after being stimulated by TGF-β1, will move from the ciliary membrane to the ciliary base and CP. Similarly, blocking the formation of the clathrin-coated pit (CCP) and clathrin-coated vesicle (CCV) by dynasore can also impair the phosphorylation and subsequent nuclear translocation of Smad2/3, and inhibit osteogenesis (Clement et al., 2013). Thus, the TGF-β signaling is highly associated with primary cilia.

Besides, as a part of the TGF-β superfamily, BMPs play an important role in bone healing. In the canonical signaling pathway, BMP ligands first bind to the BMPRII (such as ACVR2A and ACVR2B) and phosphorylate BMPRI (such as ALK−1, −2, −3, and −6). Then, intracellular BMP effector proteins Smad1, Smad5, and Smad8 are phosphorylated. Like TGF-β signaling, further transcription of canonical BMP signaling depends on the binding of Smad1/5/8 with Smad4, which then translocates into the cell nucleus to regulate gene transcription (Rahman et al., 2015). In 1998, it was reported that PEMF at 15 Hz promoted the expression of BMP2 and BMP4 in osteoblasts (Bodamyali et al., 1998). Further study demonstrated that PEMF promote osteogenesis by activating BMP-Smad1/5/8 signaling (Yan et al., 2015). Moreover, it was reported that PEMF (50 Hz, 0.6 mT) could promote the expression of BMPRIB and BMPRII in the membrane of primary cilia, and activated canonical BMP signaling to enhance osteogenesis, but IFT88 siRNA treatment abolished the PEMF induced osteogenic ability (Xie et al., 2016). Furthermore, the combination of PEMF (75 Hz, 1.5 mT) and BMP2 also showed a strong synergistic ability in promoting osteogenic differentiation of hMSCs through the canonical BMP signaling pathway (Martini et al., 2020). Take together, TGF-βs/BMPs play a central role in PEMF induced bone healing.

### 5.2 The interaction between TGF-β and BMP, and the implications in the treatment of bone fracture

The biphasic effect between TGF-β and BMP plays a fundamental role in the elaborated bone dynamics during bone healing. Immediately after the bone fracture, TGF-β is released and activated at the fracture site, which leads to a robust increase in the concentration of TGF-β. The higher concentration of TGF-β brings benefits to the recruitment and proliferation of MSCs and osteoprogenitors. The major aim of the early stage is to get a sufficient cell number basis at the local fracture site, thus the BMP signaling is inhibited due to the high concentration of TGF-β. The interaction of TGF-β and BMP is dose-dependent, and TGF-β1 at a high concentration (20 ng/ml) showed negative influences on the osteogenic ability of BMP9 (Li et al., 2012). Further study clarified that the high concentration of TGF-β1 could inhibit BMP2 by changing its biding site directly, and upregulate the expression of tomoregulin1 (Temff1) to suppress the BMP signaling indirectly (Xu et al., 2020).

As the concentration of TGF-β decreases, TGF-β1 exhibits positive effects on BMP signaling. At this stage, the major aim is to promote matrix mineralization and new bone formation; therefore, lots of energy and resources are regulated to promote the osteogenic ability. Thus, cell proliferation ceases, matrix mineralization initiates, and the primary cilia start to perceive the extracellular signals to regulate the healing process. A recent study demonstrated that TGF-β1 at a lower concentration (1 ng/ml) could activate Smad3, and enhanced the expression of BMP2 by promoting the combination of Smad3 and the BMP promoter (Xu et al., 2020). TGF-β1 at a low concentration (5 ng/ml) has a synergistic effect with BMP9 in inducing bone formation (Li et al., 2012). Moreover, TGF-β1 was able to prolong the duration of BMP2 activated Smad1/5/8 signaling, thus enhancing osteogenesis (Asparuhova et al., 2018).

Many fracture patients suffer from the complex relationship between TGF-β and BMP. Since the excessively high concentration of TGF-β exist in patients with delayed union or nonunion, BMP2 has no effect on 36% of patients with fractures (Ehnert et al., 2012). Considering the complex relationship between TGF-β and BMP in bone healing, it may be more effective and feasible to optimise EMF with the spatiotemporal specificity in promoting bone healing. Within a few days after a bone fracture, we use the EMF with a TGF-β signaling specific parameters to promote the migration and proliferation of MSCs and osteoprogenitors at the fracture site. After that, we change the EMF parameters according to a BMP specific one that activates BMP signaling to promote osteogenesis.

### 5.3 Crosstalk with other signaling pathways

**FIGURE 3:**
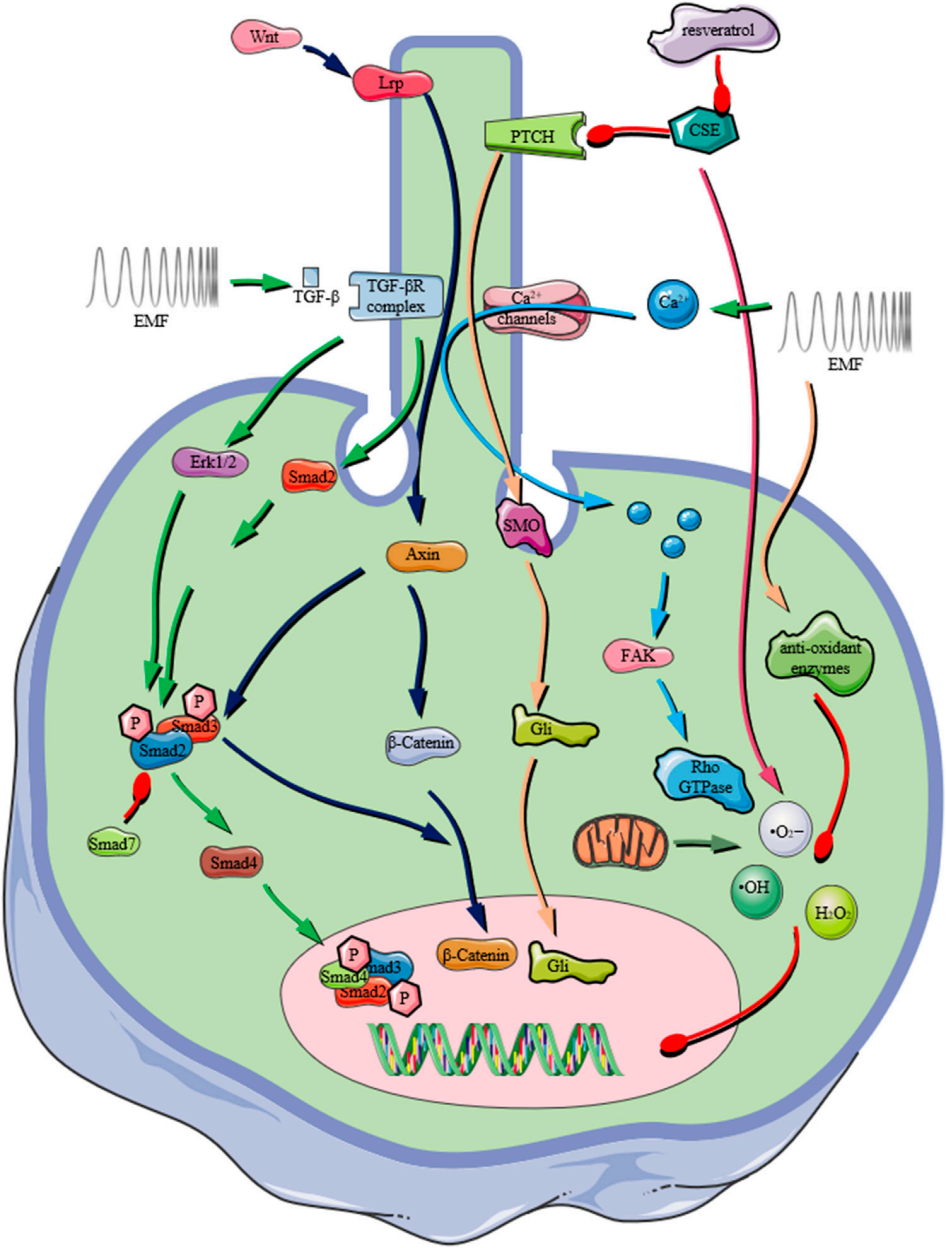
Schematic representation of the important role that the primary cilium plays in receiving extracellular physical/chemical stimuli and transducing them by various signaling pathways, *e.g.*, TGF-β signaling pathway, Wnt/β-Catenin signaling pathway, Ca^2+^ influx mediated signaling, ROS production. In recent years, PEMF exposures were reported to activate various signaling pathway depending on the presence of primary cilium on osteoprogenitors (The graphic components of the figure were provided by smart.servier.com).

For both TGF-β/BMP signaling pathways, they have extensive crosstalk with other signaling networks. For example, the activation of BMPs involves the phosphorylation of almost 400 proteins (Kim et al., 2009). Therefore, it is necessary to understand the influences of EMF on the crosstalk between TGF-β/BMP signaling and other signalings (Figure 3).

**FIGURE 4:**
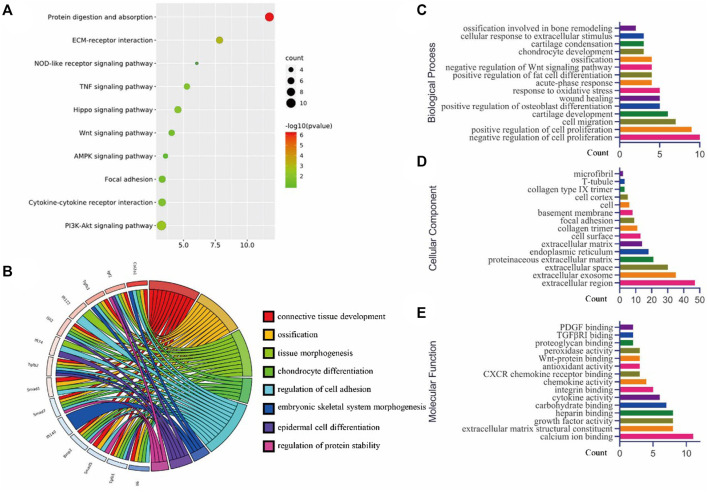
Bioinformatics analysis (GSE17825) for the top 250 genes (according to *p*-values) were analyzed using DAVID for **(A)** KEGG pathway, **(B)** the chord plot for some key genes and related GO terms, **(C)** the biological processes of GO analysis, **(D)** the cellular component of GO analysis, and **(E)** the molecular function of GO analysis (figures were plotted by https://www.bioinformatics.com.cn (last accessed on 30 July 2022), an online platform for data analysis and visualization).

Moreover, we explored the deeper mechanism for post bone fracture by analysing the gene array (Series GSE17825) from the GEO database. To have an insightful understanding of the interaction between these differentially expressed genes, we analyzed the KEGG pathways. The results indicated that different signaling pathways, such as Hippo signaling, Wnt signaling, AMPK signaling, among others, were involved in the bone healing between 1-day and 5-day post fracture (Figure 4A). The relationships between key genes and gene ontology (GO) terms are shown in the chord plot (Figure 4B). The GO terms of the biological process (Figure 4C), cellular component (Figure 4D), and molecular function (Figure 4E) were summarized.

#### 5.3.1 Wnt signaling

Wnt signaling plays a pivotal role in the development and maintenance of stem cells. The Wnt ligand can bind to the receptor Frizzled, then Lrp5 or Lrp6 interacts with Axin. After that, the function of GSK-3β is blocked after the formation of the Dishevelled- Axin-GSK-3β complex. As a result, the stabilised β-catenin is able to regulate gene transcription (Nusse, 2012). Nevertheless, Wnt signaling has an extensive crosstalk with TGF-β signaling, and both signaling pathways have synergistic effects in regulating cellular differentiation (Nusse, 2012). For example, Axin can promote the phosphorylation of Smad3, and Smad3 facilitates the nuclear entrance of β-catenin (Furuhashi et al., 2001). Jing et al. (2014) demonstrated PEMF (15 Hz, 2.4 mT) was excellent therapy for both disuse osteoporosis of hindlimb unloaded rats and ovariectomy-induced osteoporosis, by regulating Wnt/Lrp5/β-Catenin signaling *in vivo* (Jing et al., 2013). Recently, their group proved PEMF is the effective treatment for improving bone mass and the microarchitecture of the glucocorticoids-induced osteoporosis, through protecting the canonical Wnt/β-Catenin signaling impaired by the glucocorticoids (Cai et al., 2020a). Similarly, sinusoidal EMF (50 Hz, 1.8 mT) was also proven to contribute to enhancing bone mass through upregulating Wnt10b/β-Catenin signaling in a primary cilia-dependent manner (Zhou et al., 2019). Another study showed that PEMF (15 Hz, 2.0 mT) could increase serum levels of OCN, P1NP, and upregulate the gene expression of alkaline phosphatase (ALP) and Runx2 *in vivo*, through activating the canonical Wnt/β-catenin signaling (Cai et al., 2020b). Moreover, ELF-PEMF (50 Hz, 20 mT, 30 min/day) can promote the osteogenic ability of adipose-derived MSCs by increasing the expression of Wnt1, Wnt3a, β-Catenin, and Lrp5 (Fathi and Farahzadi, 2017). Interestingly, the influences of PEMF on bone healing is age-dependent. It exhibited strong positive effects in 5-month rats, while showing nearly no impact on 20-month rats (Cai et al., 2020b).

#### 5.3.2 Notch signaling

Notch signaling is activated following the binding of a ligand (such as Jagged and Delta) with the Notch receptor, and release of the Notch intracellular domain (NICD). Then NICD translocates into the nucleus to regulate Notch target genes. However, while they are important signaling in controlling cell proliferation and differentiation, Notch signaling and TGF-β/BMP signaling have extensive crosstalk and exhibit biphasic effects on bone healing. For example, the osteogenic ability of BMP can be enhanced by the ligands of Notch signaling (Tezuka et al., 2002), or inhibited by Notch1 siRNAs (Nobta et al., 2005). However, Notch signaling can also impair BMP signaling, because the overexpression of NICD produces Hey1, which impairs osteogenesis by suppressing Runx2 (Zamurovic et al., 2004). A study showed that PEMF (75 Hz, 1.5 mT) could activate canonical Notch signaling to promote the osteogenic ability of hMSCs *via* enhancing the expression of Notch ligand Dll4, Notch 4 receptor, as well as the nuclear target genes Hey1, Hes1, and Hes5 (Bagheri et al., 2018).

#### 5.3.3 Hedgehog signaling

Hh signaling is an evolutionarily conserved signaling pathway that controls tissue development and regeneration. The Hh signaling pathway is regulated after the binding of Hh ligands to the Patched receptor (PTCH). Afterwards, the smoothened (SMO) is released and move to primary cilia and activate the glioma-associated oncogene homolog (Gli). Then, Gli is translocated into the nucleus to regulate gene expression.^84^ Therefore, the primary cilium exerts distinct effects in transducing Hh-Gli signaling during bone healing. Moreover, TGF-β can promote the expression of Gli2 in a SMO/Hh independent manner,^85^ while BMP can inhibit the expression of SMO and Gli1 (Rios et al., 2004).

IFT is also involved in Hh signaling. An IFT80 deletion blocks the canonical Hh signaling pathway, and promotes the non-canonical Hh-Gαi-RhoA-ROCK signaling pathway to damage osteogenesis (Yuan et al., 2016). The knockout of IFT 140 resulted in the impaired matrix deposition *via* inhibiting the expression of PTCH1, SMO, and Gli1-3 (Li G. et al., 2018). In addition, kruppel-like factor 4 (KLF4), a transcription factor can repress osteogenesis by blocking the Hh signaling pathway (Takeuchi et al., 2018). In our previous study, we demonstrated that the cigarette smoke extract (CSE) damaged MSCs osteogenesis by impairing primary cilia and inhibiting Hh-Gli2 signaling. Meanwhile, resveratrol protected the CSE-induced dysfunction of the primary cilium and Hh-Gli2 signaling, and therefore, rescued the osteogenic ability (Sreekumar et al., 2018).

#### 5.3.4 Calcium ion channels

Ca^2+^ is an important second messenger in various physiological and pathological reactions *e.g.*, cell proliferation, metabolism, differentiation, and apoptosis (Mellstrom et al., 2008; Zhou et al., 2022). Therefore, PEMF may affect many cellular activities by reorienting charged biomolecules such as Ca^2+^ (Yan et al., 2022). It was reported that PEMF has the ability to elicit Ca^2+^ oscillation and increase the concentration of intracellular Ca^2+^ to regulate osteogenesis (Petecchia et al., 2015). In addition, a recent study reported that EMF (15 Hz, 1 mT) exhibited a lasting impact on promoting BMSCs proliferation, osteogenic ability both *in vitro* and *in vivo*, but the mechanism was unclear (Tu et al., 2018). It is plausible that Ca^2+^ channels play an important role in the legacy effects of EMF because Ca^2+^ channels can maintain the inward current to a longer process physiologically. To investigate the signaling of Ca^2+^ induced-cellular activities, recent research focused on the non-selective Ca^2+^ channel piezo1 (Wang B. et al., 2022; Liu et al., 2022). Interestingly, in our unpublished work, we proved that piezo1-induced Ca2+ influx in osteoprogenitors is highly related with the improved fracture healing, the pharmaceutically blocking piezo1 impaired the fracture healing improved by PEMF exposure (Yangmengfan Chen et al., 2022). Moreover, cell migration is also an important factor in promoting wound healing, especially for orthopaedic surgery. A recent study showed that the EMF (50 Hz, 1 mT) could promote the migration of hMSCs by increasing intracellular Ca^2+^ and enhancing FAK/Rho GTPase pathways (Zhang et al., 2018). However, a conflicting study reported that the mechanosensory ability of primary cilia was proven to be a Ca^2+^ channel-independent because bone cells without primary cilia also show Ca^2+^ flux under fluid flow. Therefore, further study should be performed to determine the role of primary cilia in Ca^2+^ related signaling transduction.

### 5.4 Reactive oxygen species

ROS, a crucial second messenger, play a pivotal role in regulating cellular metabolism. ROS consists of hydrogen peroxide (H_2_O_2_), hydroxyl radical (•OH), superoxide anion (•O_2_^−^), and hypochlorous acid (HOCl) (Wang and Zhang, 2017). These free radicals can widely affect downstream signaling, from which H_2_O_2_ can sensitively reacts to extracellular stimuli and transfuse cellular membranes to elicit a transduction cascades (Sheppard et al., 2022). Nevertheless, in high concentrations, ROS can induce strong cytotoxicity to damage cellular membranes, proteins, and DNA (Zeng et al., 2022). As a result, excessive ROS will impair the normal cellular function, structure (Yin et al., 2016), and harm the process of fracture healing (Wang K. et al., 2022). Recent studies showed that the close relationship between EMF and ROS (Li J. et al., 2018). EMF (50 Hz, 2 mT, 6 h/day) was reported to stimulate the generation of ROS (Chen et al., 2014). The same result was also reported under the treatment of 7.5 Hz EMF (Tang et al., 2016). In addition, it is interesting that the high expression of ROS can return to the base levels if the EMF exposure time is prolonged (Tang et al., 2016). However, there are some opposing results showed that 15 and 20 Hz EMF can reduce ROS generation caused by ischemia-reperfusion injury (Ma et al., 2013). The conflicting results might result from the different EMF parameters, cell types, and exposure strategy. In our previous study, the single exposure to ELF-PEMF (16 Hz) generated excessive oxidative stress on human osteoblasts, while the repetitive exposure to ELF-PEMF not only reduced the ROS level by activating various anti-oxidant enzymes but also promoted the cells viability and osteogenic ability (Ehnert et al., 2017). Therefore, applying EMF with proper parameters and exposure strategy could be a safe and effective therapy for modulating ROS level to regulate the process of fracture healing.

### 5.5 Other signaling pathways

There exist various serine/threonine kinases in different signaling pathways that can regulate R-Smads in the TGF-β/BMP signaling. JAK-STAT signaling is important in modulating cell proliferation, differentiation, and apoptosis. After ligands bind, the JAK kinases are phosphorylated and dimerized to phosphorylate and activate STAT. However, STAT3 was reported to inhibit the integration of Smad3/4 and nucleus translocation *via* competitive combination with Smad3 (Wang et al., 2016). NF-κB signaling is highly related to arthritis, and chronic inflammation. TGF-β1 can promote IκB, and inhibit NF-κB signaling that activated by tumour necrosis factor-α (TNF-α) (Monteleone et al., 2004). In turn, NF-κB activated by proinflammatory cytokines that can upregulate Smad7 to suppress TGF-β signaling (Bitzer et al., 2000). Hippo signaling is conserved signaling, which regulates the development and homeostasis of various organs, and tissue. Both YAP and TAZ in Hippo signaling are reported to be modulated by the mechanotransduction (Dupont et al., 2011). Therefore, the Hippo signaling pathway might involve in the primary cilia-related signal transduction. In addition, YAP can regulate TGF-β signaling by binding to Smad1 (Alarcon et al., 2009), Smad7 (Ferrigno et al., 2002), and modulating the translocation of Smad2/3/4 complexes (Varelas et al., 2008). The MAPK pathway also has extensive crosstalk with TGF-β signaling. For example, TGF-β can promote the function of Erk and p38 (Takekawa et al., 2002); however, Erk enables the inhibition of BMP signaling by blocking the nuclear translocation of Smad1 and promoting the degradation of Smad1 through eliciting sequential phosphorylation (Sapkota et al., 2007; Eivers et al., 2009). The sinusoidal EMF (15 Hz, 1 mT) can promote AP activity, the expression of BMP2, Runx2, and OCN significantly by phosphorylating p38 and Erk1/2 (Yong et al., 2016). The PI3K-AKT pathway has complex crosstalk activities with TGF-β signaling. For example, after strong activation of PI3 signaling, Erk is suppressed, while GSK-3 β and β-catenin are activated. Then, Smad2/3 stimulates the expression of Nanog, which is involved in the self-renewal. Meanwhile, modest activation of PI3K signaling promotes Smad2/3 to regulate mesendoderm differentiation (Singh et al., 2012). Recently, it was reported that EMF (15 Hz, 1 mT) could promote osteogenesis by promoting the ion channel P2X7, then activating Akt/GSK-3 β/β-catenin signaling *in vitro* and *in vivo* (Zhang Y. C. et al., 2019). IFT80 depletion also impairs the formation of mineralization significantly by blocking the expression and crosstalk between Hh-Gli signaling and FGF2-FGFR1-PI3K-Akt signaling (Yuan et al., 2019).

## 6 The clinic significance

**FIGURE 5:**
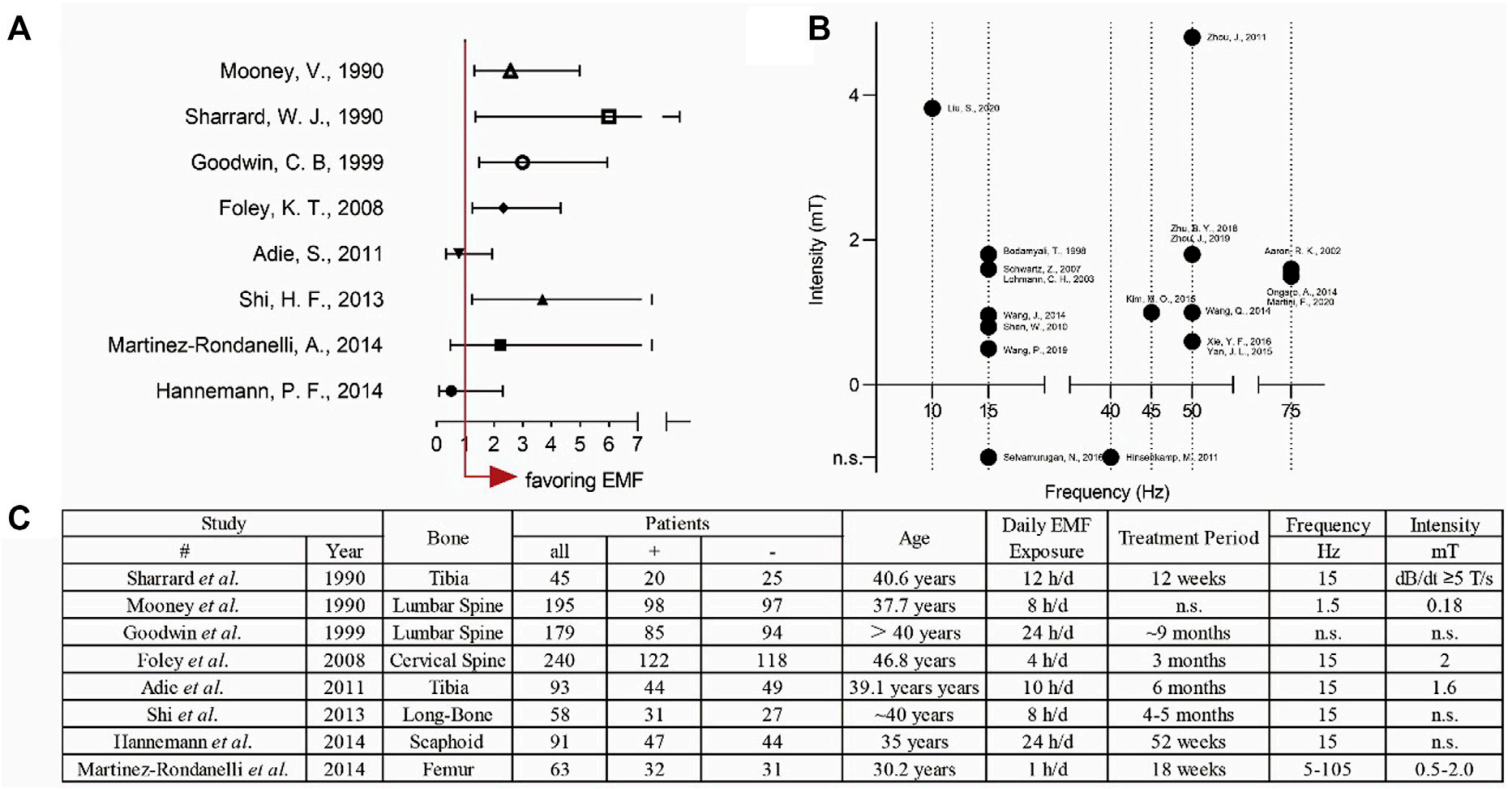
Overview of both clinical and experimental applications of EMFs. **(A)** Forest plot for analysing the clinical treatment of EMFs with odds ratio (OR) ± 95% confidence interval **(B)** Scatter plot for summarising the parameters used in the basic experiments. **(C)** The detailed data for the clinical application of EMFs.

The PEMFs were widely reported for their excellent ability to accelerate fracture healing and shorten the recovery time. We summarized the clinical cases that using ELF-PEMFs to treat bone fractures. The results indicated that ELF-PEMFs were an effective treatment for various types of bone fractures (Figures 5A,C). Recent studies showed out of 44 patients suffering from delayed bone union, or nonunion, 77.3% (34 patients) achieved clinical bone union after being treated by the PEMFs (the median treatment duration was 29.5 weeks) (Assiotis et al., 2012). The average bone healing time can be shorted from 14.7 to 8.9 weeks after the PEMFs treatment (Streit et al., 2016). In a recent prospective clinical study that involved 43 women with postmenopausal osteoporosis, PEMFs (16–22 Hz, 3–3.6 mT, 50 min/day) enable the increased expression of bone-specific AP, and decrease expression of bone resorption marker CTX by activating Wnt/β-catenin signaling (Catalano et al., 2018). In our previous study, ELF-PEMF (16 Hz, 6–282 μT, 7 min/day for 30 days) was used to treat 37 patients after high tibial osteotomy, and the results showed ELF-PEMF treatment could promote osseous consolidation, especially for those patients older than 50 years of age (Ziegler et al., 2019). However, there also exist many difficulties in evaluating the clinical effects of PEMFs, such as the inconsistency of fracture types, surgery impacts, PEMF treatment, and even the criteria for fracture union.

## 7 Outlook and discussion

With the deepening of EMFs-related research, the influences of EMFs signals on DNA raised a concern about the safety in clinical treatment. For example, EMFs can affect stem cells during early embryonic development, reduce the rate of successful assisted reproductive technologies, and increase the incidence rates of various congenital malformations for newborns, such as Wiedemann syndrome and Angelman syndrome (Maher et al., 2003). In fact, the structure of DNA is broken only under EMF with energetic radiofrequency or ionizing frequency. The EMFs with extremely low frequency are safe because this frequency can only regulate gene expression and protein synthesis (Blank and Goodman, 2009).

Primary cilia are of great importance in the development of various tissues and organs. The mutation of ciliary genes will result in more than 30 ciliopathies (Reiter and Leroux, 2017). As a manifestation of ciliopathy disorders, bone dysplasia present in many diseases such as Jeune syndrome, Ellis-van Creveld syndrome, and orofacial digital syndrome (Yuan and Yang, 2015). The Jeune syndrome mice model established by IFT140 knockout showed dwarf phenotypes with decreased bone length, density, growth retardation, and disappearance of the growth plate (Tao et al., 2019). The mutation of IFT88 resulted in attenuated mandibular development and damaged osteogenesis by inhibiting Hedgehog signaling (Kitamura et al., 2020). Considering the positive effects of ELF-EMFs on primary cilia, it is theoretically plausible that ELF-EMFs could be an effective therapy for ciliopathy disorders in the future.

In regard to the clinical efficacy of EMFs, there exist a nonlinear effect of EMF on cell activities. Chen et al. compared the influences of different intensities of PEMFs (0.6–3.6 mT, 50 Hz) on osteoblasts, the results showed all intensities of PEMF promoted cell proliferation, from which the lowest intensity (0.6 mT) was significantly higher than other intensities (Yan et al., 2015). In addition, 0.6 mT PEMF showed the best pro-osteogenic ability, while 2.4 mT PEMF exhibited almost the same ability as the control (Yan et al., 2015).

Even though many studies demonstrated that ELF-PEMFs could promote the new bone formation effectively (Ehnert et al., 2015), it is still difficult to reach the best curative effect in the clinic, because of the lack of consistency in EMFs-related research. These inconsistencies could be classified as follows:

1) The EMF generating devices are different. The generating devices consist of either a single solenoid coil or a pair of Helmholtz coils. Besides, the size, component, and structure of the compartment can also affect the EMF signals.
2) Different laboratories choose different EMF parameters, such as frequency, intensity, and exposure time. This phenomenon often leads readers to speculate that the parameters were random, which makes it difficult for researchers to improve the efficacy by modulating the parameters. Therefore, it is necessary to fix variates and figure out the best parameters (Ross et al., 2015). We summarized the EMFs frequency and intensity used in studies that related to bone healing. The result demonstrated that the most popular frequency is 15 and 50 Hz; besides, most of the intensity is lower than 4 mT (Figure 5B). This conclusion could shed light on further study to explore the effective parameters.
3) The type and components of the culture medium are different and may lead to the inconsistency. For example, the content of fetal bovine serum ranges from 0.1% to 20%.
4) Our previous study demonstrated that ELF-PEMFs could effectively promote the formation of new bone, especially when the osteogenic ability was suppressed (Ehnert et al., 2015). Moreover, excessive exposure not only showed no positive influences on bone healing but also damaged cell activity. PEMF was demonstrated to promote cellular proliferation for these cells in the proliferation stage, while stimulating the matrix formation in the differentiation stage. In addition, the inconsistencies result from the position, dimension of culture plate on the EMF generating devices, as well as cell densities in the plate.
5) The process of bone healing is a continuous activity without a cut-off point for total union. Therefore, it is necessary to have uniform criteria to define the bone union (Bhandari et al., 2012).
6) One of the advances of using EMF to treat bone nonunion is the cost-effectiveness; thus, it is better to analyze the healthcare cost, and consider the socioeconomic burden, such as increasing the EMF efficacy, and shorting the exposure duration (Button et al., 2009).

In summary, this review elucidated the important functions and insightful signaling pathways of EMFs and primary cilia on the bone healing process, which has essential clinical value and research implications for the development and optimization of EMFs for treating bone fractures in the future.

## Glossary

OH: hydroxyl radical
ACTs: activins
ACVR: activin A receptor
ALK: activin receptor-like kinase
ALP: alkaline phosphatase
BB: basal body
BMPs: bone morphogenic proteins
CCPs: clathrin-coated pits
CCVs: clathrin-coated vesicles
CDE: clathrin-dependent endocytosis
CiPo: ciliary pocket
CSE: cigarette smoke extract
CV: ciliary vesicles
DIC: differential interference contrast
ECM: extracellular matrix
EF: electronic field
PEMF: extremly low frequency-pulsed electromagnetic field
EMF: electromagnetic field
Erk: extracellular signal-regulated kinase
Gli: glioma-associated oncogene homolog
H_2_O_2_: hydrogen peroxide
Hh: hedgehog
HOCl: hypochloric acid
Hz: herz
IFT: intraflagellar transport
INHs: inhibins
KLF4: kruppel-Like Factor 4
LLC: large latent TGF-β1 complex
LTBP: latent TGF-β binding protein
MAPK: mitogen-activated protein kinase
MF: magnetic field
MMPs: matrix metalloproteinases
MSCs: mesenchymal stem cells
NF-κB: nuclear factor-κB
NICD: notch intracellular domain
OCN: osteocalcin
OSX: osterix
OVX: ovariectomy
PC1: polycystin-1
PDGF: platelet-derived growth factor
PEMF: pulsed electromagnetic field
PTCH: patched receptor
RANKL: receptor Activator of NF-κB Ligand
ROS: reactive oxygen species
Runx2: runt-related transcription factor 2
SLC: small latent TGF-β complex
SMO: Smoothened
TEP: transepithelial potential difference
TGF-β: transforming growth factor-β
TGF-β R: TGF-β receptor
TNF-α: tumor necrosis factor-α
TZ: transition zone
